# Mn oxide formation by phototrophs: Spatial and temporal patterns, with evidence of an enzymatic superoxide-mediated pathway

**DOI:** 10.1038/s41598-019-54403-8

**Published:** 2019-12-03

**Authors:** Dominique L. Chaput, Alexandré J. Fowler, Onyou Seo, Kelly Duhn, Colleen M. Hansel, Cara M. Santelli

**Affiliations:** 1 Department of Mineral Sciences, National Museum of Natural History, Smithsonian Institution, Washington, DC USA; 2 Biosciences, University of Exeter, Exeter, UK; 3 Department of Earth and Environmental Sciences, University of Minnesota - Twin Cities, Minneapolis, MN USA; 4 Marine Chemistry and Geochemistry, Woods Hole Oceanographic Institution, Woods Hole, MA USA

**Keywords:** Element cycles, Element cycles, Element cycles

## Abstract

Manganese (Mn) oxide minerals influence the availability of organic carbon, nutrients and metals in the environment. Oxidation of Mn(II) to Mn(III/IV) oxides is largely promoted by the direct and indirect activity of microorganisms. Studies of biogenic Mn(II) oxidation have focused on bacteria and fungi, with phototrophic organisms (phototrophs) being generally overlooked. Here, we isolated phototrophs from Mn removal beds in Pennsylvania, USA, including fourteen Chlorophyta (green algae), three Bacillariophyta (diatoms) and one cyanobacterium, all of which consistently formed Mn(III/IV) oxides. Isolates produced cell-specific oxides (coating some cells but not others), diffuse biofilm oxides, and internal diatom-specific Mn-rich nodules. Phototrophic Mn(II) oxidation had been previously attributed to abiotic oxidation mediated by photosynthesis-driven pH increases, but we found a decoupling of Mn oxide formation and pH alteration in several cases. Furthermore, cell-free filtrates of some isolates produced Mn oxides at specific time points, but this activity was not induced by Mn(II). Manganese oxide formation in cell-free filtrates occurred via reaction with the oxygen radical superoxide produced by soluble extracellular proteins. Given the known widespread ability of phototrophs to produce superoxide, the contribution of phototrophs to Mn(II) oxidation in the environment may be greater and more nuanced than previously thought.

## Introduction

Manganese (Mn) oxide minerals are ubiquitous in soils and sediments. Due to their exceptional scavenging and redox capabilities, Mn oxides exert a disproportionately high influence on the availability and fate of organic carbon (e.g. lignin, humic acids), nutrients (e.g. phosphate) and metals (e.g. Pb, Zn, Co, Ni, As, Cr) in the environment^1–5^. The formation of these minerals can occur via abiotic pathways in systems with elevated pH^6^, but in most natural environments, it is thought to be induced by the direct and/or indirect activity of Mn(II)-oxidising microorganisms^3,7^.

Due to the importance of Mn oxides in the environment, and given that dissolved Mn is a problematic pollutant in many aquatic systems, notably in mining and other industrial drainage^8–10^, there is great interest in isolating Mn-oxidising microbes and elucidating the mechanisms involved. Most studies have focused on bacteria and fungi, generally assumed to be the main contributors to the oxidation of Mn(II) to Mn oxides^3,7,11^. For bacteria and ascomycete fungi, Mn(II) oxidation is not coupled to growth or energy conservation, and the physiological basis for Mn(II) oxidation remains elusive. In contrast, basidiomycete fungi oxidise Mn(II) to Mn(III), which then oxidises recalcitrant carbon such as lignin^12,13^. Several enzymatic pathways are involved in microbial Mn(II) oxidation, including multicopper oxidases used by some fungi and bacteria^7,14–18^, manganese peroxidase used by white-rot fungi^12,19,20^, and superoxide-mediated pathways employed by some bacteria^21–23^ and ascomycete fungi^24,25^. Enzymatic pathways are often induced only under specific environmental conditions^23^ and during discrete developmental phases^18,24^, and they can be triggered by microbe-microbe interactions^26^.

Regardless of the pathway or microbial catalyst, the oxidation of Mn(II) to Mn(IV) occurs via two independent one-electron transfer processes involving the formation of a Mn(III) intermediate^14,22,27–31^. Indeed, recent methodological advances have indicated that soluble Mn(III) complexes represent a dominant component of the dissolved Mn pool within oxic^32–34^, suboxic^32,35,36^, and anoxic^32,37–40^ systems. The Mn(III) ion is unstable and will undergo disproportionation, but dissolved organic carbon (DOC) can form Mn(III)-ligand complexes (Mn(III)-L) that stabilise Mn(III). Thus, the cycling and distribution of Mn is dependent upon the concentration and composition of DOC ligands. The reaction pathway from Mn(III) to Mn(IV) is less clear and may involve either disproportionation to Mn(II) and Mn(IV) or further oxidation to Mn oxides either through a second electron transfer reaction within the enzyme^16^ or by the same or secondary oxidant. Reaction between Mn(II) and superoxide leads to the formation of Mn(III) and hydrogen peroxide (H_2_O_2_), another ROS. In the absence of stabilising ligands, Mn(III) and H_2_O_2_ can rapidly back-react to (re)generate Mn(II), inhibiting the formation of Mn oxides^41^.

Phototrophs have largely been overlooked in studies of Mn(II)-oxidising organisms. Yet, the oxygen-evolving complex of photosystem II (PSII) contains four redox-active Mn atoms that cycle between oxidation states to accumulate oxidising capacity, linking the single-electron pigmented reaction centres to the four-electron oxidation of two water molecules to O_2_^42,43^. Drill cores through an early Paleoproterozoic succession (2.415 Ga) contain Mn enrichment that predates the rise of molecular oxygen, suggesting that the oxygen-evolving complex of PSII arose from an earlier photosystem that carried out single-electron oxidation of Mn and formed Mn oxide minerals rather than O_2_^43,44^. Thus, Mn oxidation lies at the heart of photosynthesis; it is directly responsible for most of Earth’s primary productivity, and at least partially responsible for the increase in free O_2_ in the atmosphere^42^.

It has long been observed that phototrophs can precipitate Mn oxide minerals directly. Diatoms were considered the initiators of some Mn concretions as early as 1932^45^, and in natural waters, a strong correlation was noted between natural phytoplankton blooms and increased amounts of particulate Mn^46^. Pure culture research confirmed Mn(II) oxidation by phototrophs^47–51^, though this process was primarily attributed to pH increases that occur when phototrophs consume dissolved CO_2_. Homogeneous abiotic Mn(II) oxidation by O_2_ to form Mn(IV) oxide minerals is not favoured under standard environmental conditions (pH < 8) due to a reactivity barrier at the first electron transfer^52^. The reaction becomes favourable (albeit slow) around pH 8, accelerating above pH 9 and becoming autocatalytic above pH 10 (with 1 atm O_2_)^52^. Phototrophs can generate sufficiently high pH microenvironments for abiotic Mn(II) oxidation to proceed^46^, and some authors have proposed using the presence of Mn oxides around individual algal cells and aggregates as an indicator of high-pH microenvironments^48^.

The widespread assumption that phototrophs precipitate Mn oxides only indirectly via this photosynthesis-linked pH increase does not appear to have been challenged directly, beyond some experiments showing that pH buffering compounds, growing phototrophs in the dark, or adding the PSII inhibitor DCMU all inhibit Mn(II) oxidation^47,48,53^. However, some authors have noted that photosynthesis-linked pH-mediated oxidation does not fully explain their observations; for example, an alga isolated from acidic soil could form Mn oxides at pH 5.0^54^, and in a Mn(II)-contaminated creek, only some diatoms became coated with Mn oxides, despite all diatoms presumably carrying out photosynthesis^55,56^. More recently, Mn(II) oxidation has been linked to extracellular superoxide production by several bacteria^22,23^ and fungi^24,25^. A wide range of phototrophic organisms, including diatoms, cyanobacteria, and algae, produce extracellular superoxide for reasons that remain poorly understood^57–63^. Superoxide diffusion across the cell membrane is limited^64^ and thus extracellular levels originate from the univalent reduction of O_2_ by trans-membrane or outer-membrane associated enzymes^65,66^. Thus, it is possible that Mn(II) oxidation by some phototrophs may proceed via similar superoxide-mediated pathways.

Here, we undertook a culture study in a Mn-impacted environment that we previously characterised by culture-independent amplicon sequencing^67^ to (i) obtain phototrophic isolates tolerant of high Mn(II) concentrations, (ii) determine which, if any, of the phototrophs could oxidise Mn(II) to produce Mn oxides, and (iii) elucidate whether observed Mn oxide formation was solely due to photosynthesis-driven pH increases, or whether there were other operative oxide formation pathways.

## Results and Discussion

### Diversity of Mn(II)-oxidising phototrophs

We found that Mn oxide formation activity was widespread among phototrophs isolated from two manganese removal beds (MRBs) treating coal mine drainage in western Pennsylvania, De Sale Phases 1 and 2 (DS1 and DS2, respectively). Ninety-eight isolates were obtained from the enrichment flasks inoculated with algal mats or crushed Mn oxides (see Supplementary Information for detailed culturing conditions), and all showed some degree of Mn oxide formation, confirmed both by visible observation of brown/black particulates and by leucoberbelin blue (LBB), which turns a deep blue colour in the presence of Mn(III,IV) oxides^68^. It is possible that Mn(III)-ligand complexes were also formed (the reaction of LBB with Mn(III)-ligand complexes is more nuanced and depends largely on the nature of the ligand^69^), but these complexes were not directly quantified. No growth or Mn oxide formation was observed after several months in the enrichment flasks inoculated with water samples, which suggests that the majority of culturable phototrophs in the MRBs are biofilm-forming rather than planktonic. The absence of Mn oxides in these water-inoculated flasks also shows that the culture media alone (including the buffer TRIS in MBL medium) do not oxidise Mn(II).

We selected eighteen isolates for further study, as they all consistently produced Mn oxides and covered a broad taxonomic range (Table 1). The selected isolates included one cyanobacterium, three diatoms (phylum Bacillariophyta), and fourteen green algae (phylum Chlorophyta) from two classes: Chlorophyceae (orders Sphaeropleales, Oedogoniales and Chlamydomonadales) and Trebouxiophyceae (order Chlorellales) (Table 1, Table S1). Isolate names beginning with ‘CM’, ‘MB’ and ‘WC’ were isolated and maintained in COMBO, MBL and WC media, respectively, and for clarity, isolate names are followed by .gr, .cy or .di for green alga, cyanobacterium and diatom, respectively.

**Table 1:** Taxonomy, Mn(II) tolerance and Mn(II) oxidation activity of phototrophs isolated from CMD passive treatment systems.

Isolate	Consensus taxonomy^a^	Mn(II) tolerance (mM)	Oxidation in unbuffered media solid/liquid^b^	Oxidation in buffered liquid media^c^	Oxidation by cell-free extract^b^
CM7-6	Chlorophyta: Chlorellaceae	≥10	−/+	−	+
CM8-1	Chlorophyta: *Scenedesmus* sp.	2	(+)/+	pH 6.0-8.0	+
CM8-2	Bacillariophyta: *Seminavis* sp.	0.5	−/+	pH 6.5-8.0*	−
CM8-5	Chlorophyta: *Scenedesmus obliquus*	2	+/+	−*	nt
CM8-6	Chlorophyta: unknown Trebouxiophyceae	≥10	−/+	−	−
CM9-5	Chlorophyta: unknown Chlorophyceae	≥10	−/+	pH 8.0	−
CM9-6	Chlorophyta: unknown Chlorophyceae	5	+/+	−*	−
CM11-1	Chlorophyta: unknown Chlorophyta	2	+/+	−*	nt
CM11-2	Chlorophyta: unknown Oedogoniales	1	−/+	pH 5.5-8.0	−
CM12-1	Chlorophyta: unknown Scenedesmaceae	≥10	(+)/+	pH 7.0-8.0	+
CM12-4	Bacillariophyta: *Nitzschia palea*	2	−/+	pH 7.0-8.0*	−
CM12-5	Chlorophyta: *Chlorella* sp.	≥10	+/+	−*	−
MB7-1	Chlorophyta: unknown Chlorophyta	2	−/+	pH 5.5-8.0	−
MB7-4	Bacillariophyta: *Nitzschia palea*	≥10	−/+	pH 6.5-8.0	−
MBx9-1	Cyanobacteria: *Pseudanabaena* sp.	≥10	+/+	−*	+
WC6-3	Chlorophyta: *Chlamydomonas* sp.	2	(+)/+	pH 8.0	+
WC7-3	Chlorophyta: unknown Chlamydomonadales	≥10	+/+	−*	nt
WC8-1	Chlorophyta: *Oedogonium* sp.	2	+/+	−	(+)

^a^Consensus from multiple markers (plastid 23S rRNA, 18S rRNA, rbcL), listed in Table S1.

^b^Minus sign indicates no oxidation, (+) indicates inconsistent oxidation, +indicates consistent oxidation, nt means ‘not tested’.

^c^Minus sign indicates no oxidation at any pH, listed pH values indicate ranges with Mn(II) oxidation, *indicates growth inhibition by one or more buffers.

The filamentous cyanobacterial isolate MBx9-1.cy (the only cyanobacterium among the original 98 isolates) was placed in the genus Pseudanabaena, which is common in aquatic and benthic environments^70^ but has no known Mn(II)-oxidising members. However, its class, Oscillatoriophycideae, contains two reported Mn(II)-oxidising members, *Microcystis* sp.^47,48^ and *Oscillatoria* sp.^71^, though the latter was not an axenic culture but rather the dominant member of a mixed microbial mat used for bioremediation of Mn-contaminated mine drainage. The 23S rRNA sequence from MBx9-1.cy was present in the environmental phototroph-specific amplicon pyrosequencing data previously obtained from DS2^67^, but at low abundance, 0.06%, and it was not detected in sequence data from DS1, despite having been isolated from that MRB.

Numerous Mn(II)-oxidising diatoms were obtained from the enrichment flasks (35 of the initial 98 isolates), but only three were selected for further study, as the rest were extremely sensitive to antibiotics (making it difficult to obtain axenic cultures) and most stopped growing after several months, pointing to deficits in our culture conditions. The plastid 23S rRNA fragment of the diatom CM8-2.di most closely resembled *Seminavis robusta*, whereas diatom isolates CM12-4.di and MB7-4.di most closely resembled *Nitzschia palea*, at 100% similarity (Table S1). Plastid 23S rRNA is too conserved in diatoms to serve as a high-resolution barcode^72^, but an 18S rRNA sequence obtained from CM12-4.di also resembled *Nitzschia palea*, at 99.7% similarity. Of all the isolates, the diatoms were the most abundant in the phototroph amplicon sequencing data^67^, with CM8-2.di accounting for 2.5% of sequences from DS1 at 97% similarity and over 10% of sequences in both MRBs at 95% similarity (Table S1). SEM of sediments from MRBs in Pennsylvania (USA) showed freshwater diatoms in close proximity to Mn oxides^73^. Light microscopy of glass slides submerged in a Mn-contaminated creek in Arizona, USA^55,56^ and in high-Mn water samples from Quadrilatero Ferrifero, Brazil^74^ showed Mn oxide accumulation on diatoms. A pure culture of the diatom *Nitzschia* sp. oxidised Mn(II), but only within aggregates and not on single cells^48^, leading those authors to conclude that high pH microenvironments resulting from photosynthesis were the sole mechanism of oxidation.

Of the fourteen green algal isolates, CM7-6.gr and CM12-5.gr belonged to the Chlorella clade (genera Micractinium and Chlorella, respectively), microalgae with worldwide distribution that are known to withstand environmental stress and are used in food and biofuel production^75,76^. Mn(II) oxidation has previously been reported by Chlorella isolates obtained from a freshwater lake^47,48^. Two isolates, CM8-1.gr and CM8-5.gr, were placed in the genus Scenedesmus, with a third isolate, CM12-1.gr, belonging to the parent family Scenedesmaceae (though it could not be classified further). Like Chlorella, the genus Scenedesmus shows worldwide distribution in all climates^77^. Pure Scenedesmus cultures from a freshwater lake and from a culture collection^48,49^ have shown Mn(II)-oxidising activity, as has a relative in the family Scenedesmaceae, *Desmodesmus* sp. WR1, isolated from raw municipal wastewater^51^. Other isolates were from genera with widespread distribution in freshwater and other environments but not previously known to have Mn(II)-oxidising members: unbranched filamentous green algae Oedocladium (CM11-2.gr) and Oedogonium (WC8-1.gr), and unicellular green algae Chlamydomonas (WC6-3.gr) and Chlorococcum (WC7-3.gr). Five isolates (CM8-6.gr, CM9-5.gr, CM9-6.gr, CM11-1.gr, MB7-1.gr) had no close relatives in GenBank or had conflicting results from different marker genes (Table S1). Of all the green algal isolates, those in the family Oedogoniales (CM11-2.gr and WC8-1.gr) were the most abundant in the environmental amplicon data, accounting for 1.45% of DS1 sequences. The others were present at relative abundances below 1% or could not be detected at all (Table S1).

### Growth and Mn oxide formation patterns

**Figure 1:**
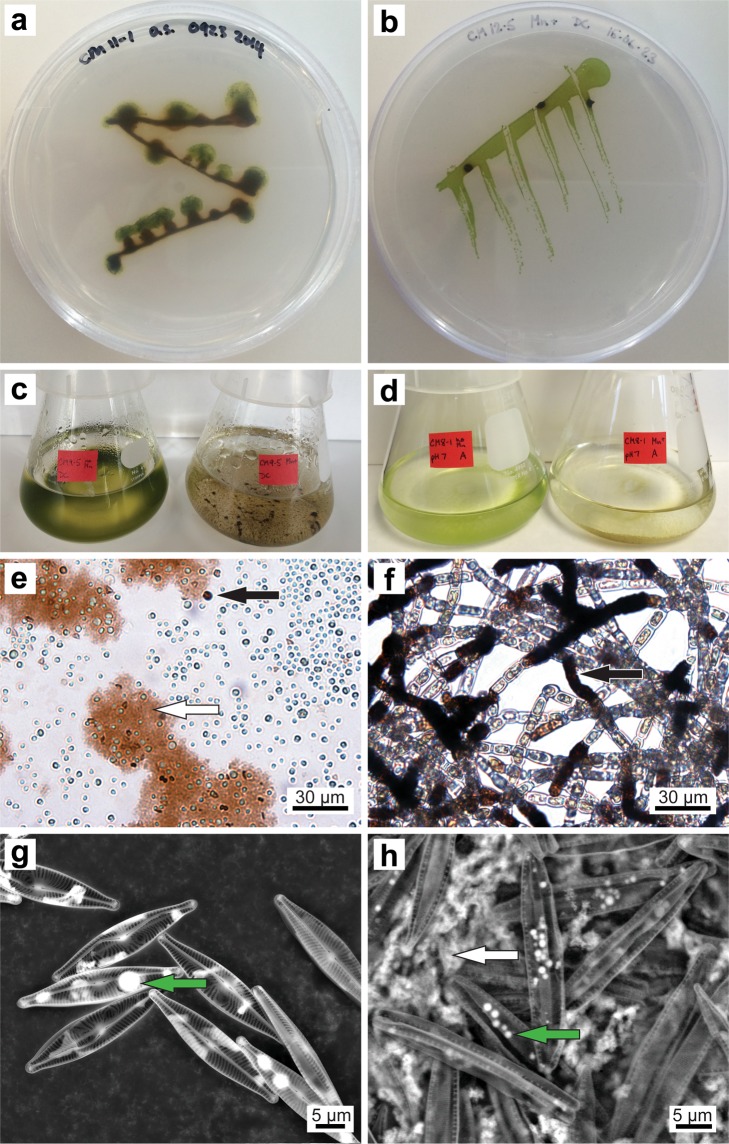
Examples of Mn(II) oxidation by phototrophs. Mn oxides appear as brown/black precipitates (**a**–**f**) and as bright white precipitates in SEM images (g,h). (**a**) CM11-1.gr on solid Mn+ COMBO after 86 days. (**b**) CM12-5.gr on solid Mn+ COMBO after 56 days. (**c**) CM9-5.gr in liquid COMBO after 56 days (left = Mn-free, right = Mn+). (**d**) CM8-1.gr in liquid COMBO with 10 mM HEPES pH 7, after 20 days (left = Mn-free, right = Mn+). (**e**,**f**) Bright-field microscopy of glass slides submerged in Mn+ COMBO. White arrow shows diffuse oxidation throughout the biofilm, black arrows indicate cell wall-associated oxidation. (**e**) CM7-6.gr after 15 days, (**f**) WC8-1.gr after 15 days. (**g**,**h**) SEM of diatoms CM8-2.di after 29 days and CM12-4.di after 37 days, respectively. Intracellular Mn-rich nodules are present with both isolates (green arrows), and white arrow (**h**) shows diffuse biofilm-associated oxidation.

Similar to bacterial and fungal cultures obtained from these same field sites^78^, the isolates were tolerant of high Mn(II) concentrations, exceeding 10 mM in many cases (Table 1). With most green algal isolates, the presence of Mn(II) in the culture media resulted in a slower growth rate and, interestingly, biofilm growth rather than the planktonic form observed in Mn-free media (Fig. 1c). Exceptions to this lifestyle difference included the two filamentous Oedogoniales isolates CM11-2.gr and WC8-1.gr, which remained planktonic, as well as the three diatoms and the cyanobacterium *Pseudanabaena* sp. MBx9-1.cy, which were biofilm-forming even in the absence of Mn(II). Biofilm growth requires copious production of extracellular polymeric substances (EPS). High concentrations of dissolved Mn(II) have been shown to change the quantity and composition of EPS produced by some bacteria^79,80^, and the presence of Mn oxides could also be modifying the characteristics of EPS through breakdown, polymerisation or stabilisation reactions^6^. EPS have often been the site of biogenic Mn oxide accumulation in bacteria, algae and fungi^7,55,56,81,82^. EPS could promote Mn(II) oxidation by serving as adsorption and nucleation sites, by allowing the development of steep pH and O_2_ gradients, and by concentrating metabolites and enzymes excreted by cells. Furthermore, Mn oxides, such as birnessite, may induce the polymerization of low molecular weight organic carbon^6^.

**Figure 2:**
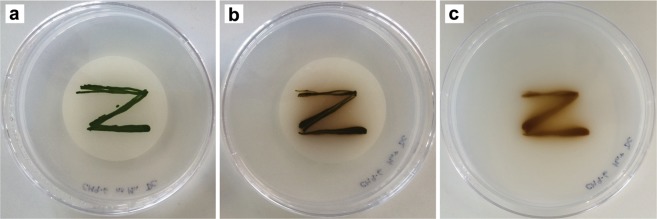
Mn(II) oxidation on agar-solidified media by single-celled, non-motile green algal isolate CM9-6.gr. CM9-6.gr was grown for 64 days on a 0.2 μm filter placed on the surface of agar-solidified COMBO. (**a**) Mn-free medium; (**b**) Mn+ medium, with brown/black Mn oxide precipitates visible within the algal colonies and extending across the filter surface. (**c**) Same plate as in panel (**b**) after removal of the filter containing algal colonies, showing Mn(II) oxidation directly beneath the colonies and extending 1-2 cm outwards.

All isolates produced Mn oxides readily when grown in liquid culture, but only seven showed consistent oxide formation activity on agar-solidified media (Table 1). The patterns of Mn(II) oxidation on solid media varied, with some isolates precipitating Mn oxides within distinct areas of the colonies (Fig. 1a,b) or in the media immediately below the colonies, and others showing diffuse Mn oxides forming within the media and away from the colonies (Figs. 1a and 2). The non-motile green algal isolate CM9-6.gr (unknown Chlorophyceae) exhibited the most rapid and extensive Mn oxide formation activity on solid media; the zone of Mn oxide deposition extended 1-2 cm into and across the agar plate, even when the cells were grown on a 0.2 μm filter placed on the surface of the agar (Fig. 2). In liquid culture, light microscopy of glass slides incubated in the flasks showed two different patterns of Mn oxide formation, both of which were present with most isolates (Fig. 1e,f, Supplementary Fig. S1) and in the initial enrichment flasks (Supplementary Fig. S2). First, Mn oxides were seen coating some cells but not others (referred to hereafter as ‘cell-specific oxidation’). Second, Mn oxides were observed in diffuse clumps throughout areas of the biofilm, either overlying large groups of cells or not obviously associated with any cells in particular (referred to as ‘diffuse biofilm oxidation’). SEM further demonstrated both cell-specific and diffuse biofilm Mn oxide precipitation (Fig. 1h, Supplementary Fig. S3). It also showed a third type of Mn accumulation, in intracellular nodules specific to diatoms (Fig. 1g,h, Supplementary Fig. S3e–h), although it was not determined whether these nodules were Mn oxides or another Mn-rich phase, due to their atypical morphology. The different growth and oxidation patterns observed in the presence of Mn(II) may stem from its role as an essential nutrient for phototrophs (notably in PSII) but its toxicity at high concentrations. The growth of phototrophs is inhibited at low Mn concentrations, so they are particularly good at scavenging and concentrating this metal^49,83,84^. The Mn-rich nodules observed inside diatoms could be a by-product of this capability, though Sunda and Huntsman^84^ found that hyper-accumulation of Mn did not occur when two diatom species in the genus Thalassiosira were exposed to Mn concentrations that exceeded their intracellular transport capacity. However, given that Mn is toxic to many phototrophs at high concentrations^49,85^, protection against toxicity is a more likely explanation for the changes in growth form and perhaps some of the oxidation patterns we observed. For example, biofilm growth would provide an EPS barrier between the cells and culture medium, and diffuse biofilm oxidation may be a means of lowering the concentration of dissolved Mn within the matrix.

**Figure 3:**
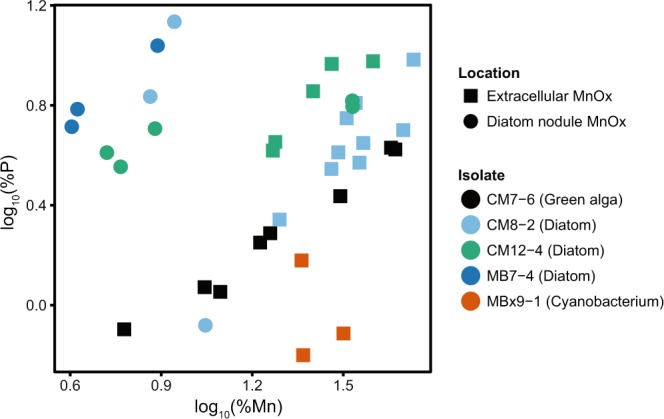
Relative abundance of P versus Mn in extracellular Mn oxides and in intracellular Mn-rich nodules, measured by energy-dispersive X-ray spectroscopy. Isolates beginning with ‘CM’ were grown in COMBO medium, and those beginning with MB were grown in MBL medium (Table S2).

A curious feature of the Mn oxides produced by the phototrophs was the co-occurrence of phosphorus (Fig. 3, Supplementary Figs. S4, S5), which was enriched in the extracellular Mn oxides produced by all three diatoms and by the green alga CM7-6.gr (*Chlorella* sp.), and especially abundant in the diatom intracellular Mn nodules. The cyanobacterial (MBx9-1.cy) extracellular Mn oxides did not exhibit the same degree of phosphorus enrichment despite being grown in the same medium (MBL) as one of the diatoms (MB7-4.di). The two growth media (COMBO and MBL) differ mainly in their trace elements and the presence of TRIS in MBL; they have identical amounts of phosphate and of most other essential nutrients (Table S2), so the growth medium is unlikely to be solely responsible for this difference. To our knowledge, phosphorus enrichment in biogenic Mn oxides has not been reported with bacterial or fungal pure cultures. It is unclear whether the phosphorus we observed was due to adsorption of phosphate from the culture media onto the Mn oxides^86^, whether it was from a separate biological substance (e.g. a component of EPS and/or a phospholipid bilayer surrounding the diatom intracellular nodules), or whether it was incorporated within the Mn mineral structure and possibly forming a mineral phase other than Mn oxide. Examples of microbial precipitation of other Mn-rich phases include the Mn-reducing bacterium *Shewanella putrefaciens*, which can precipitate dissolved Mn(II) as Mn(II) phosphate, Mn_3_(PO_4_)_2_^87^, and a marine microbial consortium grown in Mn(II)-amended media, shown to produce brown Mn precipitates that included the expected Mn(III/IV) oxides but also MnCO_3_ and Na_3_Mn(PO_4_)(CO_3_), both Mn(II) compounds^88^. All the phototroph cultures used for EDS, including the diatom MB7-4.di that had intracellular nodules but no visible extracellular Mn-rich material, were LBB-positive, indicating the presence of Mn(III/IV), but it is possible the brown precipitates that we had assumed were solely Mn(III/IV) oxides also contained some Mn phosphate compounds.

### Can photosynthesis-driven pH increase alone explain all phototrophic Mn(II) oxidation?

Homogeneous oxidation of Mn(II) by O_2_ is thermodynamically and kinetically favourable above pH 8, accelerating with increasing pH and becoming autocatalytic above pH 10^52,89^. As such, Mn oxidation by phototrophs had previously been attributed to pH increases caused by the consumption of dissolved CO_2_ and the resulting removal of carbonic acid, either in the bulk water or in microenvironments surrounding algal cells^46–48,56^.

Some of our findings were consistent with this pH-mediated abiotic O_2_ oxidation mechanism. The bulk pH of the initial enrichment flasks at the time of termination (after 4 months) was elevated for most media types: AF6 = 9.33 ± 0.88, BB = 9.98 ± 0.60, CM = 8.93 ± 0.36 and WC = 8.25 ± 0.23 (n = 10, mean ± SE), from a starting pH of 6.6 to 7.8, depending on the media type (Table S2). However, MBL flasks, all of which had extensive deposition of Mn oxides, had a mean pH of 7.61 ± 0.13 (n = 10). Pure cultures of numerous isolates also raised the pH of unbuffered liquid media after three months, in some cases well above pH 8.0: CM8-1.gr (9.72), WC8-1.gr (9.2), CM7-6.gr (8.96), CM8-6.gr (8.86), CM12-1.gr (8.70). Adding Good’s buffers to the media, which restricted bulk pH increases to +0.3 pH units, stopped Mn oxide formation altogether with nine of the isolates, and those maintained in MBL medium (buffered with TRIS) showed much more Mn oxide formation when they were instead grown in one of the unbuffered media types (COMBO or WC). Phototrophs, however, are known to be sensitive to pH buffering compounds, including TRIS and phosphate^90^. In our buffered experiments, many isolates showed reduced growth rates when MES, MOPS or HEPES were present in the media (Table 1), even when the buffer concentration was reduced from 10 mM to 1 mM and the starting pH was identical to the unbuffered control. The inhibition of Mn oxide formation within media containing pH buffers could therefore be due to a physiological response to the buffering compounds and not to the pH buffering itself. Alternatively, in some cases at least, Mn(II) oxidation could have proceeded to Mn(III) followed by stabilisation of Mn(III) by the organic buffer, prohibiting the formation of Mn oxides. The Mn(III) complexation behaviour of the buffers used here is currently unknown, though Mn(II)-oxidising bacteria and fungi have produced Mn oxides in some of these buffers previously^5,78,91,92^.

However, many of our other findings were not consistent with a pH-driven mechanism. Although the isolates raised the pH of unbuffered bulk media over time, Mn oxides invariably appeared in culture flasks before the pH reached 8.0 (Supplementary Fig. S6). Of course, measurements of bulk media pH do not take into account pH microenvironments surrounding cells or within biofilms, which can be considerably different from the bulk liquid^46,48^. Light microscopy and SEM showed some diffuse biofilm-associated Mn oxides, which could be due to pH gradients within the biofilm matrix. However, cell-specific Mn oxides are more difficult to explain with a purely pH-driven abiotic O_2_ oxidation mechanism. Only some cells in a rapidly growing culture were coated by Mn oxides despite all the cells presumably carrying out photosynthesis and thus having an elevated pH surrounding the cell (Fig. 1e,f, Supplementary Figs. S1–S3), an observation that mirrors those of Robbins *et al*.^55^ and Robbins and Corley^56^ with diatoms on glass slides submerged in a Mn-contaminated creek. This pattern was especially noticeable with the filamentous isolate *Oedogonium* sp. WC8-1.gr (Fig. 1f), which had Mn oxide-coated cells adjoining oxide-free cells along the same filament. Cell-specific oxide formation was observed with most isolates, including those smaller than 20 μm. Richardson and Stolzenbach^48^ presented empirical evidence and mathematical models showing that pH gradients sufficiently large for abiotic Mn(II) oxidation by O_2_ can only form around individual cells or aggregates larger than 20 μm, so at least in some cases, pH gradients alone may not entirely explain Mn oxide formation by our isolates. Knauer *et al*. came to a similar conclusion in their study of a Mn-oxidising pure culture of the green alga *Scenedesmus subspicatus*; neither cell-generated pH gradients nor Mn oxidation autocatalysis could explain their observed rates of Mn oxidation^49^. In our study, when pH buffers were added to the media, nine isolates continued to show Mn oxide formation over a wide range of pH values (Fig. 1d, Table 1), sometimes as low as 5.5. This oxidation activity would imply steep gradients over small distances for abiotic, high pH oxidation by O_2_ to be the sole mechanism. An isolate of the green alga *Chlorella* sp. was shown to generate pH gradients from 7.5 to 10.3^47^, so gradients over 2-3 pH units are possible, but pH-mediated oxidation in media buffered to 5.5 would require a more extreme gradient of 4-5 pH units.

**Figure 4:**
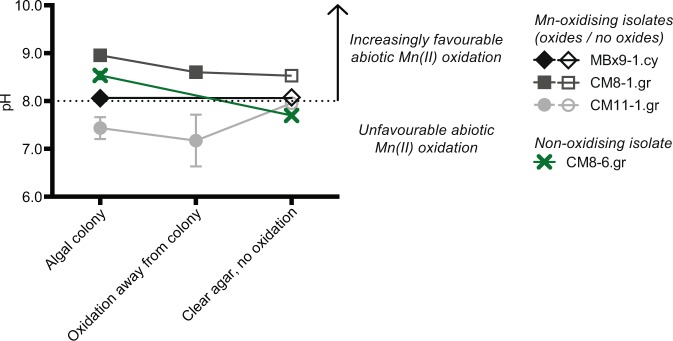
pH microprobe measurements of cyanobacterial and green algal isolates growing on agar plates with 200 μM Mn(II). CM8-6.gr (green cross) showed no Mn oxidation, MBx9-1.cy had oxidation within the colonies only, whereas CM8-1.gr and CM11-1.gr had oxidation both within the colonies and in the agar surrounding the colonies. pH measurements were taken within the colonies, in areas of oxidation surrounding colonies (where present), and in clear agar with no visible oxidation. Symbols show mean pH, error bars show range (n = 2 biological replicates, each consisting of 3 technical replicate measurements). Filled symbols indicate the presence of Mn oxides, empty symbols indicate no Mn oxides.

Furthermore, pH microenvironments surrounding cells could not account for the diffuse Mn oxide formation observed with some isolates on agar plates, where Mn oxides were deposited in the agar far from the cell colonies (Fig. 2). Mn oxidation becomes autocatalytic at high pH (above pH 10), but it is unlikely that small colonies could change the pH of such a large area of an agar plate by CO_2_ consumption alone, and maintain it at that level, when the plate has an extensive surface area for atmospheric CO_2_ equilibration. Indeed, pH microprobe measurements across agar plates confirmed that areas with Mn oxides did not have consistently higher pH than clear areas of agar (Fig. 4). Contrary to the findings of Richardson *et al*.^47^ and Lubbers *et al*.^46^ in liquid cultures, the pH inside the colonies growing on agar was not consistently elevated, ranging from a low of 7.21 in the Mn(II)-oxidising isolate CM11-1.gr, to a high of 8.96 in Mn(II)-oxidising isolate CM8-1.gr. Internal colony pH did not appear correlated with the presence of Mn oxides - the second-highest colony pH values (8.46-8.61) were in an isolate that did not produce Mn oxides when grown on agar (CM8-6.gr), whereas the isolate CM11-1.gr produced oxides both within its colonies and in the surrounding agar, despite maintaining a pH well below 8 in both these areas (Fig. 4). Instead, as observed previously for fungi^25^, these types of Mn oxide deposits can occur in two ways: (1) Mn(II) is oxidised to Mn(III)-L at the cell surface and then transported from the cell where it then is further oxidised or disproportionates to Mn oxides within the agar, or (2) a soluble enzyme/metabolite is secreted and oxidises Mn(II) to Mn(III) and ultimately Mn oxides within the agar at a distance from the cells.

The combination of Mn oxide formation over a wide pH range, cell specific Mn oxide formation within cultures, and decoupling of lower pH micro-environments and Mn oxide precipitates on plates suggest that mechanisms other that elevated pH are primarily responsible for Mn oxide formation within these cultures.

### A more complex phototrophic Mn(II) oxidation pathway

Since photosynthesis-linked pH increases alone failed to explain Mn oxide formation in buffered media, cell-specific Mn oxide accumulation patterns, and diffuse Mn oxide deposition far from colonies on agar plates, we conducted a series of experiments (i) to identify other parameters required for Mn oxides to form, notably the age of the culture and whether Mn(II) is required for the oxidation pathway to be induced, and (ii) to identify essential components of the phototrophic Mn oxide formation pathway – specifically, whether there is involvement of proteins or the oxygen radical superoxide.

Cell-free filtrates (CFFs) of cultures were prepared by centrifugation followed by filtration through 0.2 μm filters. Five isolates yielded CFFs that consistently produced Mn oxides, indicating that in these phototrophs, the process responsible for Mn oxide formation is not strictly cell wall-associated but instead related to a soluble factor secreted by the organism (Table 1). Oxidation activity within the CFFs indicates that small molecules and/or proteins are involved in either directly or indirectly (e.g. via superoxide production) forming Mn oxides.

**Figure 5:**
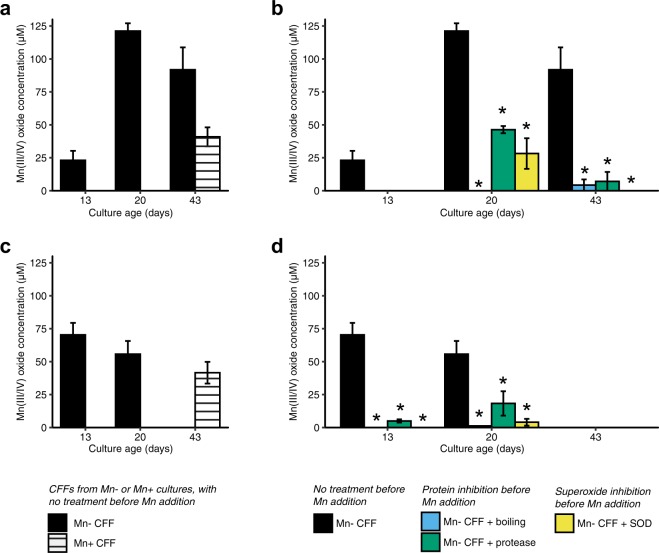
Impact of protein inhibition (boiling, protease) and superoxide inhibition (superoxide dismutase, SOD) on Mn(II) oxidation activity of algal cell-free filtrates (CFFs) prepared from cultures of different ages. CFFs were prepared from cultures with 0 μM (Mn−) or 200 μM Mn(II) (Mn+), and after treatments, Mn(II) was added to all CFFs to a final concentration of 500 μM. (**a**,**b**) *Scenedesmus* sp. CM8-1.gr, and (**c**,**d**) *Chlamydomonas* sp. WC6-3.gr. (**a**,**c**) compare Mn− and Mn + CFFs. (**b**,**d**) show the effects of boiling, protease and SOD on Mn- CFFs only [black bars are the same in (**a**,**b**) and in (**c**,**d**)]. Mn(III/IV) concentration was measured by LBB assay standardised with KMnO_4_, with a lower limit of quantification of 12.5 μM. Bars show mean Mn(III/IV) concentration, error bars show SE (n = 3). Asterisks show treatments that are significantly different from the Mn-only control, with correction for multiple comparisons (Dunnett’s test, p < 0.001).

This initial screening of CFFs was carried out at a single time point (day 60) and only with Mn + cultures (i.e. those grown in the presence of Mn(II)). More thorough experiments with isolates *Scenedesmus* sp. CM8-1.gr and *Chlamydomonas* sp. WC6-3.gr showed that CFFs prepared from Mn-free cultures had earlier and stronger Mn oxide formation activity than parallel CFFs prepared from Mn + cultures (Fig. 5a,c), indicating that the compounds responsible for the oxide formation are produced even in the absence of Mn(II), i.e. the pathway is not specifically induced by Mn(II) but rather appears to be a side reaction of unknown function, similar to previous observations for a marine bacterium within the common *Roseobacter* clade^66,93^ and several Ascomycete fungi^24,94^. The weaker oxide formation activity of the Mn + CFFs could be due to the slower growth of Mn + cultures, to the switch from planktonic to biofilm forms, to the consumption of Mn oxidants by reaction with Mn(II) prior to filtration, or to removal of Mn oxide-forming enzymes during filtration from encrustation of these enzymes by Mn oxides (as observed with the marine bacterium *Roseobacter* sp. AzwK- 3b)^5^. All Mn + flasks had copious amounts of Mn oxides prior to CFF preparation, indicating substantial oxidation activity, but compounds involved in the oxidation pathway were potentially retained within the biofilm or encrusted in the Mn oxides, whereas the Mn-free planktonic cultures were growing more quickly and secreting compounds directly into the bulk media, from which the CFFs were prepared.

Further investigations of *Scenedesmus* sp. CM8-1.gr and *Chlamydomonas* sp. WC6-3.gr revealed that Mn oxide formation activity in CFFs was time-dependent; in Mn + source culture CFFs, it was not detected with either isolate until day 43, whereas in CFFs from Mn-free source cultures, it peaked and then subsided as the culture aged (Fig. 5). CFFs from Mn-free *Scenedesmus* sp. CM8-1.gr cultures had the highest Mn oxide formation activity at day 20. CFFs from Mn-free *Chlamydomonas* sp. WC6-3.gr cultures produced Mn oxides most strongly at day 13, and by day 43 there was no detectable oxide forming activity. A similar age-dependent pattern was reported with the bacterium *Mesorhizobium australicum* T-G1^23^ and with Ascomycete fungi^94^. The observation that the concentration of Mn oxides did not simply increase over time as metabolites accumulated suggests that the changes in the Mn oxide formation activity is a function of changing composition within the secretome (both metabolites and enzymes) composition over time. Results may further suggest that Mn(III)-ligand complexes could be contributing to these time dynamics – diverse ligands with different thermodynamic and kinetic stability constants^40,95–97^ could be produced by each organism. The accumulation of Mn oxides with time could ultimately result from direct Mn(III)-ligand oxidation, similar to that observed in *Pseudomonas* sp.^98^, Mn(III) disproportionation (e.g., from pH changes that destabilise the complex or from ligand hydrolysis^99^) or fast oxidation kinetics. Indeed Qian *et al*.^99^ observed that Mn(III)-pyrophosphate disproportionation was rapidly catalysed after a time lag by Mn oxide surfaces accumulating in their experimental conditions. While the presence of Mn(III) was not measured in these experiments, our speculation along with recent studies of the importance and prevalence of soluble Mn(III) in oxic environments^33,36,37^ indicates that further investigation of Mn(III) production by phototrophs is warranted.

Boiling the Mn-free source culture CFFs or treating them with protease prior to adding Mn(II) significantly decreased or completely halted Mn oxide formation, compared with the untreated control (Dunnett’s test, p < 0.001, Fig. 5). While boiling could alter the activity of reactive dissolved small molecules, proteases are specific to protein activity. Proteins are therefore required for the formation of Mn oxides. These proteins could include enzymes that oxidise Mn(II) directly, such as multicopper oxidases that have been widely associated with Mn(II) and Mn(III) oxidation in both bacteria and fungi^7,14–17,20^. Alternatively, these could include exoenzymes that produced superoxide. NAD(P)H oxidases (NOX enzymes)^100^, which are widely known for producing superoxide within eukaryotes, are transmembrane enzymes and typically not found within secretomes of eukaryotes. Instead, glutathione reductase was recently shown to produce superoxide in the marine diatom *Thalassiosira oceanica*^62^, and haem peroxidase was shown to be the superoxide-producing enzyme in the Mn(II)-oxidising *Roseobacter* sp. AzwK-3b^66^. Lastly, within the secretome, the presence of catalase would promote Mn oxide formation by removing hydrogen peroxide, which reacts with the intermediate Mn(III), reducing it back to Mn(II)^41^.

**Figure 6:**
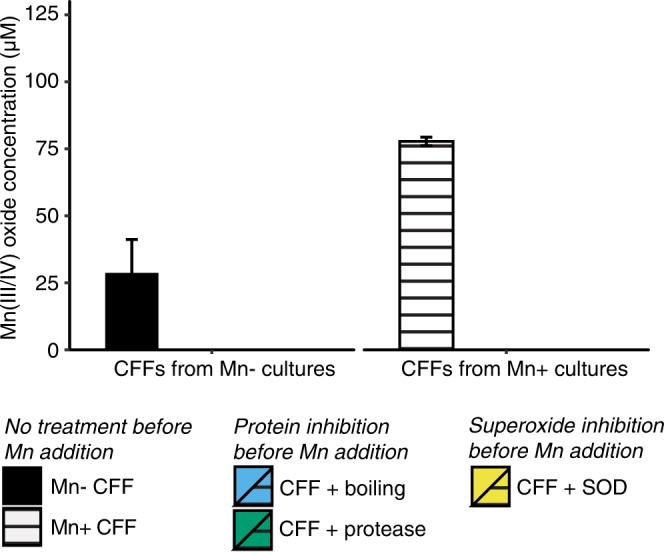
Mn(II) oxidation by cell-free filtrates (CFFs) of cyanobacterial isolate MBx9-1.cy on day 43, and the effects of protein inhibition (boiling, protease) and superoxide inhibition (superoxide dismutase, SOD) on oxidation activity. CFFs were prepared from cultures with 0 μM (Mn−) or 200 μM Mn(II) (Mn+), and after treatments, Mn(II) was added to all CFFs to a final concentration of 500 μM. Mn(III/IV) concentration was measured by LBB assay standardized with KMnO_4_, with a lower limit of quantification of 12.5 μM. Bars show mean Mn(III/IV) concentration, error bars show SE (n = 3).

Incubating the CFFs with the superoxide scavenger superoxide dismutase (SOD) prior to adding Mn(II) also significantly reduced (~80%) or halted Mn oxide formation (Dunnett’s test, p < 0.001, Fig. 5). Although the CFF experiments with multiple time points were carried out only with two green algal isolates, *Scenedesmus* sp. CM8-1.gr and *Chlamydomonas* sp. WC6-3.gr, additional experiments at a single time point (day 43) showed that the general pattern also holds with the cyanobacterial isolate *Pseudanabaena* MBx9-1.cy (Fig. 6); oxide formation is completely halted by boiling, protease and SOD treatments, suggesting a similar pathway. Any superoxide produced via cell surface or transmembrane processes (e.g. via transmembrane NAD(P)H oxidases or the photosynthetic complex, PSI and PSII) would not have been present in the CFFs due to the short lifetime of superoxide (typically < minutes). The possibility of other extracellular proteins contributing to Mn oxide formation cannot be ruled out, particularly because the addition of SOD did not fully inhibit (to 100%) oxide formation with all isolates.

In combination, the SOD and boiling/protease results indicate that Mn(II) is oxidised by superoxide that is produced by a presently unidentified soluble extracellular enzyme (or enzymes) in all of these organisms. In addition to the possibility of other extracellular proteins, there may be cell-associated oxidation pathways (e.g. multicopper oxidases, haem peroxidases), as suggested by the deposition of Mn oxides directly on specific cells of most isolates. Since the CFF experiments would have included only soluble extracellular enzymes, it is not known whether the cell-specific Mn oxide formation occurs via the same superoxide-mediated pathway or whether different processes are involved.

## Conclusion

We found that a wide diversity of phototrophs isolated from manganese bioremediation systems, including green algae, diatoms, and a cyanobacterium, promoted Mn(II) oxidation to Mn oxide minerals. The Mn oxides produced by green algae and diatoms were enriched in phosphorus, and additionally, diatoms accumulated manganese in intracellular nodules. Contrary to what has been previously reported, we found that phototrophic Mn oxide formation is not solely due to photosynthesis-linked pH increases, but rather it involves proteins and superoxide. Induction of the Mn(II) oxidation pathway does not require the presence of Mn(II), suggesting it is a side reaction of unknown function. Given the known widespread ability of phototrophs to produce superoxide^57–63^, the contribution of phototrophs to Mn(II) oxidation in the environment may be greater and more nuanced than previously thought, with repercussions on our understanding of carbon, nutrient and metal cycling.

## Materials and Methods

### Site and sample descriptions

Samples were collected in December 2012 from two Mn(II) removal beds (MRBs) of passive treatment systems in western Pennsylvania, De Sale Phases 1 and 2 (DS1 and DS2). These bioremediation systems, described previously^67,78,101,102^, contain limestone cobbles that increase solution pH and harbour Mn-oxidising microorganisms. As Mn-contaminated coal mine drainage flows through the bed, Mn(II) is oxidised and solid Mn(III/IV) oxides are retained within the beds, thus removing dissolved Mn from the waters before discharging. Site-specific details can be found in the Supplementary Information.

From each MRB, the following samples were collected: three algal mat samples, one sample of Mn(III/IV) oxide minerals scraped off a submerged limestone cobble, and two water samples from standing pools with visible algal growth on the limestone surfaces. Samples were collected with sterile plastic disposable spatulas, stored in 50 ml Falcon tubes, kept on ice during transport to the laboratory, and stored at 4 °C until inoculation (within 48 hours of collection).

### Culture enrichment, isolation and identification

Culture enrichments were initiated in 200 ml flasks containing 70 ml phototroph-specific media amended with 200 μM MnCl_2_. Six media types were used: AF6, COMBO, Bold’s Basal Medium, MBL, Bristol Medium and WC (recipes and references in Table S2). Isolates were obtained from the culture enrichments using serial dilution and streak plate approaches. Mn oxide formation by enrichments and isolates was confirmed with leucoberbelin blue (LBB), which turns a deep blue colour in the presence of Mn(III,IV) oxides^68^. Detailed methodology about culture enrichments and isolations is described in the Supplementary Information. Once isolates appeared axenic, DNA was extracted using the FastDNA SPIN Kit (MP Biomedicals). Several markers were amplified and sequenced for identification (Table S3): plastid 23S rRNA, 18S rRNA and rbcL, encoding the large subunit of RuBisCO (full details in the Supplementary Information). Putative taxonomic assignment was made at the level where there was a consensus between multiple markers.

### Light and scanning electron microscopy

Sterile glass microscope slides were placed in 300 ml flasks containing 100 ml culture media and either mixed phototrophic communities from enrichment flasks or pure isolates. Slides were carefully removed without disturbing the biofilms, mounted with coverslips, and viewed immediately using bright field microscopy.

For scanning electron microscopy (SEM), biomass was transferred to carbon tape-coated stubs and air-dried in a laminar flow hood. Images were taken on uncoated samples using a FEI Nova NanoSEM 600 variable pressure ultra-high-resolution field emission gun SEM under low vacuum using a gaseous back-scatter detector (GAD). The presence or absence of specific elements (e.g. Mn, Si, Ca, P, etc.) was confirmed using energy dispersive X-ray spectroscopy (EDS) at specific locations (individual spectra) or in large areas (elemental maps).

### Mn and pH tolerance

To assess Mn tolerance, isolates were grown in the unbuffered medium in which they were initially isolated, supplemented with a range of MnCl_2_ concentrations: 0.2, 0.5, 1.0, 2.0, 5.0 and 10.0 mM. Separate growth experiments were conducted to verify whether Mn oxide formation still occurs in buffered media, and to assess the toxicity of synthetic pH buffers, to which some algae are known to be sensitive^90^. Isolates were grown in liquid media amended with two concentrations (1 mM and 10 mM) of three different buffers (MES, MOPS, and HEPES), each at three pH values (MES 5.5, 6.0 and 6.5, MOPS 6.5, 7.0 and 7.5, and HEPES 7.0, 7.5 and 8.0), plus an unbuffered control, all with 200 μM MnCl_2_. Growth was assessed visually every two weeks for 3 months, and Mn oxide formation was first assessed visually (brown/black colouration) then confirmed with LBB. Bulk media pH was measured at the end of the experiment.

### pH microprobe measurements

To determine the pH of media during Mn oxide formation, the localised pH within and surrounding microbial colonies was measured on isolates growing on agar-solidified media supplemented with 200 μM MnCl_2_, using a micromanipulator-mounted 100 μm diameter pH microelectrode (Unisense, Aarhus, Denmark) paired with a 5 mm diameter external reference electrode (Radiometer Analytical) also placed in the media. pH measurements were taken directly inside phototroph colonies (with and without oxidation), in brown-coloured areas of Mn oxide formation away from colonies, and in clear areas with no detectable growth or Mn oxide formation. Three technical replicate measurements were made at each location and averaged.

### Cell-free filtrate oxidation experiments

Isolates were grown in triplicate 300 ml flasks containing 100 ml media with and without 200 μM MnCl_2_. At selected time points, cell-free filtrates (CFFs) were prepared as follows. Cultures were homogenised by swirling and 15 ml was decanted into sterile Falcon tubes. Cells were pelleted by centrifugation at 3000 × g for 5 minutes, and supernatants were filtered through 0.2 μm syringe filters into clean tubes. The dissolved Mn concentration (primarily Mn(II), but the presence of colloidal Mn(III/IV) can not be entirely excluded) in the CFFs from Mn + flasks was quantified with the formaldoxime assay^103^ on a Cary 60 UV–Vis spectrophotometer (Varian). The CFFs were split into five 1 ml aliquots for the following treatments: Mn-free control, Mn only control, 1 mg ml^−1^ protease (Type XIV, from *Streptomyces griseus*, Sigma, USA), 50 U ml^−1^ superoxide dismutase (SOD, Sigma, USA), and boiling. CFFs were incubated with their treatment compounds at room temperature for one hour, except the boiling treatment, which consisted of 40 minutes boiling plus twenty minutes cooling at room temperature. MnCl_2_ was added, to a final concentration of 500 μM, to all tubes except the Mn-free controls. Tubes were incubated in the dark at room temperature for one week and Mn oxides were quantified using the LBB assay (see Supplementary Information for detailed methodology).

Two-way ANOVA was used to compare treatments at each time point. Post-hoc pairwise comparisons of Mn oxide concentrations in the treatment groups versus the Mn only control were carried out with Dunnett’s multiple comparisons test, with a family significance level of 0.05. Analyses were carried out in R version 3.5.1^104^ with the package DescTools version 0.99.28^105^.

## Supplementary information

Supplementary Information

## Data Availability

All sequences from this project were uploaded to GenBank, accession numbers MF278588-MF278601 (eukaryotic plastid 23S rRNA), MF278958 (cyanobacterial 23S rRNA), MF278602-MF278609 (18S rRNA), and MF278342-MF278353 and MG787874 (*rbcL*).
