# Supplementary material for: Mn oxide formation by phototrophs: Spatial and temporal patterns, with evidence of an enzymatic superoxide-mediated pathway
Source: Sci Rep. 2019 Dec 3;9:18244. doi: 10.1038/s41598-019-54403-8 (PMC6890756; doi:10.1038/s41598-019-54403-8)
Supplement: Supplementary file 1 — Supplementary Information [file 41598_2019_54403_MOESM1_ESM.pdf]

Mn oxide formation by phototrophs: Spatial and temporal patterns, with evidence of an enzymatic superoxide-mediated pathway

Dominique L. Chaput[1,2]*, Alexandré J. Fowler[1], Onyou Seo[1], Kelly Duhn[3], Colleen M. Hansel[4], Cara M. Santelli[1,3]*

[1]Department of Mineral Sciences, National Museum of Natural History, Smithsonian Institution, Washington, DC, USA; [2]Biosciences, University of Exeter, Exeter, UK; [3]Department of Earth Science and BioTechnology Institute, University of Minnesota - Twin Cities, Minneapolis, MN, USA; [4]Marine Chemistry and Geochemistry, Woods Hole Oceanographic Institution, Woods Hole, MA, USA

*Corresponding authors:

Dominique L. Chaput, Biosciences, College of Life and Environmental Sciences, Geoffrey Pope, University of Exeter, Stocker Road, Exeter EX4 4QD, UK. Email: d.chaput@exeter.ac.uk

Cara M. Santelli, University of Minnesota, Earth Sciences Department, John T. Tate Hall-Suite 150, 116 Church St. SE, Minneapolis, MN 55455-0231, USA. Email: santelli@umn.edu

The authors declare no competing interests.

This supplementary information contains:

Supplementary Materials and Methods

Supplementary Figures S1 to S6

Supplementary Tables S1 to S3

Supplementary References

SUPPLEMENTARY MATERIALS AND METHODS

Site descriptions

Additional information on the performance of manganese removal beds (MRBs) De Sale 1 (DS1) and De Sale 2 (DS2) is available at http://www2.datashed.org/de-sale-phase-i/ and http://www2.datashed.org/de-sale-phase-ii/, respectively. At the time of sampling, DS1 had a mean influent Mn concentration of 19.9 mgL^{-1} [1]. It was largely clogged with Mn(III/IV) oxides and other sediments, impeding the flow of water, and was not attenuating any dissolved Mn(II). DS2 had a mean influent concentration of 31.2 mgL^{-1} [1] but water flow was not impeded, as limestone cobbles had been abraded and loose sediments removed approximately five years prior to sampling. It was attenuating roughly 50% of influent Mn. We previously characterised the microbial communities in these systems using amplicon pyrosequencing [2] and assessed their culturable Mn(II)-oxidizing fungi and bacteria [3].

Culture enrichment and isolation

Algal mat subsamples were combined and vortexed briefly to separate cells, yielding one pooled mat sample per MRB. Subsamples of the two water samples were combined into a single pooled water sample per MRB. Mn oxide subsamples were mixed with 10 mL sterile water and vortexed. Serial dilutions to $1/10^2$ were prepared with sterile water.

Flasks were inoculated with 50 µl of each sample and incubated at room temperature under ambient light conditions, near north-facing windows but out of direct sunlight. After one month, 10 µl media from each enrichment flask were plated onto agar-solidified media to separate and purify colonies. Substantial growth was observed on the flask surfaces rather than in the liquid, so multiple samples were taken using sterile cotton-tipped applicators and streaked onto agar-solidified media. The presence of Mn(III/IV) oxides was confirmed with leucoberbelin blue (LBB), which turns a deep blue colour in the presence of Mn(III) and Mn(IV) [4].

Colonies promoting Mn(II) oxidation were transferred to fresh liquid and agar-solidified media a minimum of three times. Mixed communities were separated by serial dilutions and streak plates. Isolates with bacterial co-cultures were grown on plates and in liquid containing ampicillin, kanamycin, penicillin or streptomycin at a range of concentrations, including on gradient plates. However, these antibiotics often proved toxic to the phototrophs, so axenic cultures could not be obtained for two isolates (CM11-1 and MBx9-1). In all other cases, removal of bacterial co-cultures with antibiotics did not change the Mn(II) oxidation activity of the phototroph.

A ~410 bp fragment of plastid 23S rRNA gene was amplified with primers AlgaeF and AlgaeR [5], cleaned with the Qiagen PCR Purification Kit, and submitted to GENEWIZ (South Plainfield, NJ, USA) for Sanger sequencing. Chromatograms were trimmed, primers were removed, and clean DNA sequences were clustered into groups with 100% similarity in Sequencher 5.4.1 (Gene Codes Corporation). Cultures consisting of a single phototroph (based on clean 23S rRNA sequencing reads) were selected, and one isolate was kept from groups with 100% sequence similarity. Cultures were maintained in liquid and on agar-solidified media containing 200 μM $MnCl_2$, at room temperature under ambient light conditions.

Following selection of the final isolates, additional markers were sequenced to support taxonomic identification: 18S rRNA with primers EK-82F/EK-1520R [6], and a fragment of the *rbcL* gene encoding the large subunit of RuBisCO (Ribulose-1,5-bisphosphate carboxylase/oxygenase), with primer pairs rbcL 7F/rbcL803 and rbcL320/rbcL1391R [7,8]. All primers used in this study are listed in Table S2. Amplicons were cleaned, sequenced and processed as described above. Sequences were searched against the GenBank database [9] using BLAST [10]. Relative abundance of plastid 23S rRNA sequences in a culture-independent sequencing survey of the two MRBs [2] was determined using BLAST.

pH tracking experiment

Most isolates increase the pH of unbuffered media over time, so to verify whether Mn oxidation occurs before the bulk media pH rises over pH 8.0, three isolates were grown in duplicate 300 ml flasks containing 125 ml COMBO media with 200 μM $MnCl_2$. pH was measured at regular intervals over 37 days, from 5 ml samples taken aseptically from the flasks (without replacement). Measurements were taken in early- to mid-afternoon to minimise the impact of diurnal variation. The appearance of Mn oxides was assessed visually.

Leucoberbelin blue (LBB) assay

Mn(III/IV) oxides produced by cell-free extracts were quantified using the LBB assay against a standard curve of freshly-made $KMnO_4$ (linear in the range 5-100 μM, R^2=0.999), with the Mn-free control as blank. Briefly, tubes were vortexed to resuspend the Mn(III/IV) oxides, and 50 μl were pipetted into 96-well plates. LBB reagent (150 μl) was added, plates were incubated in the dark for 15 minutes, and absorbance at 620 nm was read on a Synergy HT Multi-Mode Microplate Reader (BioTek Instruments, Inc., USA). Samples with measurements exceeding 100 μM (the upper end of the standard curve's linear range) were diluted and measured again. Those lower than 5 μM were deemed to be below the assay's limit of quantification (where the standard curve was no longer linear) so were assigned a value of zero. Although this assay can be adapted to quantify lower concentrations by determining a separate standard curve below 5 μM, due to the high Mn concentrations used in this research, we did not focus on the lower range but rather optimised the assay for higher levels, which resulted in a high limit of quantification. Values were adjusted using a conversion factor of 2.5, as 1 M $KMnO_4$ oxidizes 5 M LBB versus 2 M LBB oxidized by 1M Mn(IV) oxide. This assumes all the Mn in the mineral phase is Mn(IV). If, instead, it consists of a mixture of Mn(III) and Mn(IV), this approach would result in an underestimation [11].

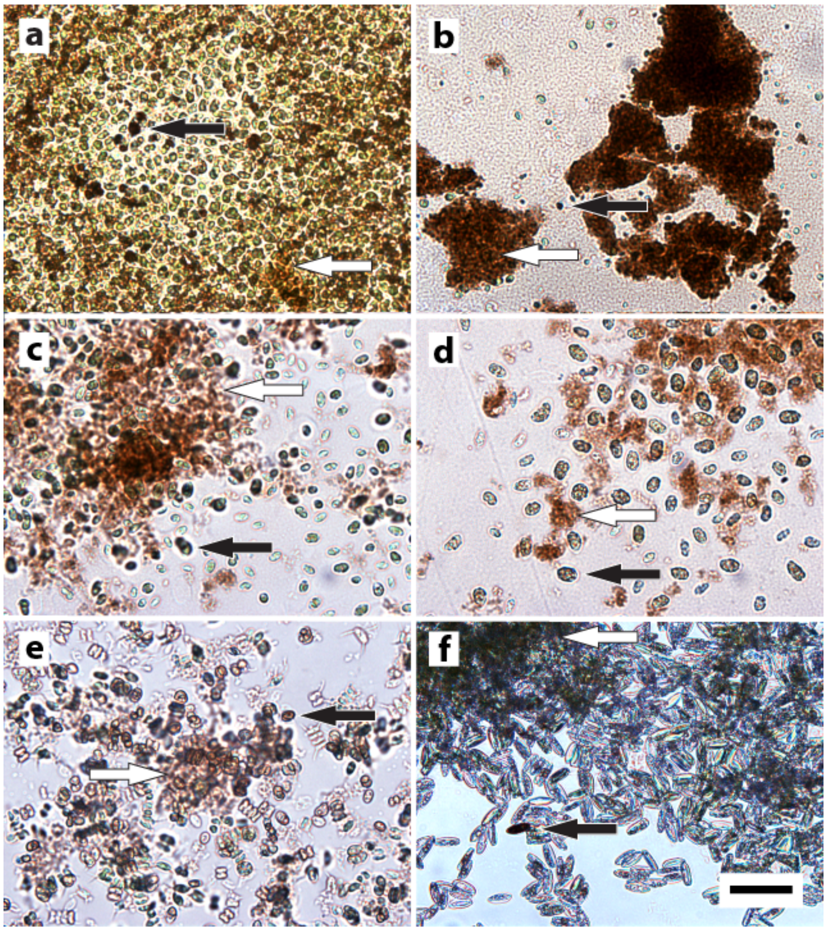

Figure S1. Bright-field microscopy of Mn(II)-oxidizing algal isolates growing on glass slides submerged in culture media containing 200 μM Mn(II). Mn oxides appear brown/black under transmitted light. Scale bar is 30 μm. White arrows indicate diffuse oxidation throughout the biofilm, and black arrows indicate cell wall-associated oxidation. (a) WC6-3.gr after 15 days. (b) CM11-1.gr after 29 days. (c) CM8-6.gr after 15 days. (d) CM9-5.gr after 15 days. (e) CM8-1.gr after 15 days. (f) CM12-4.di, an elongated diatom, after 90 days.

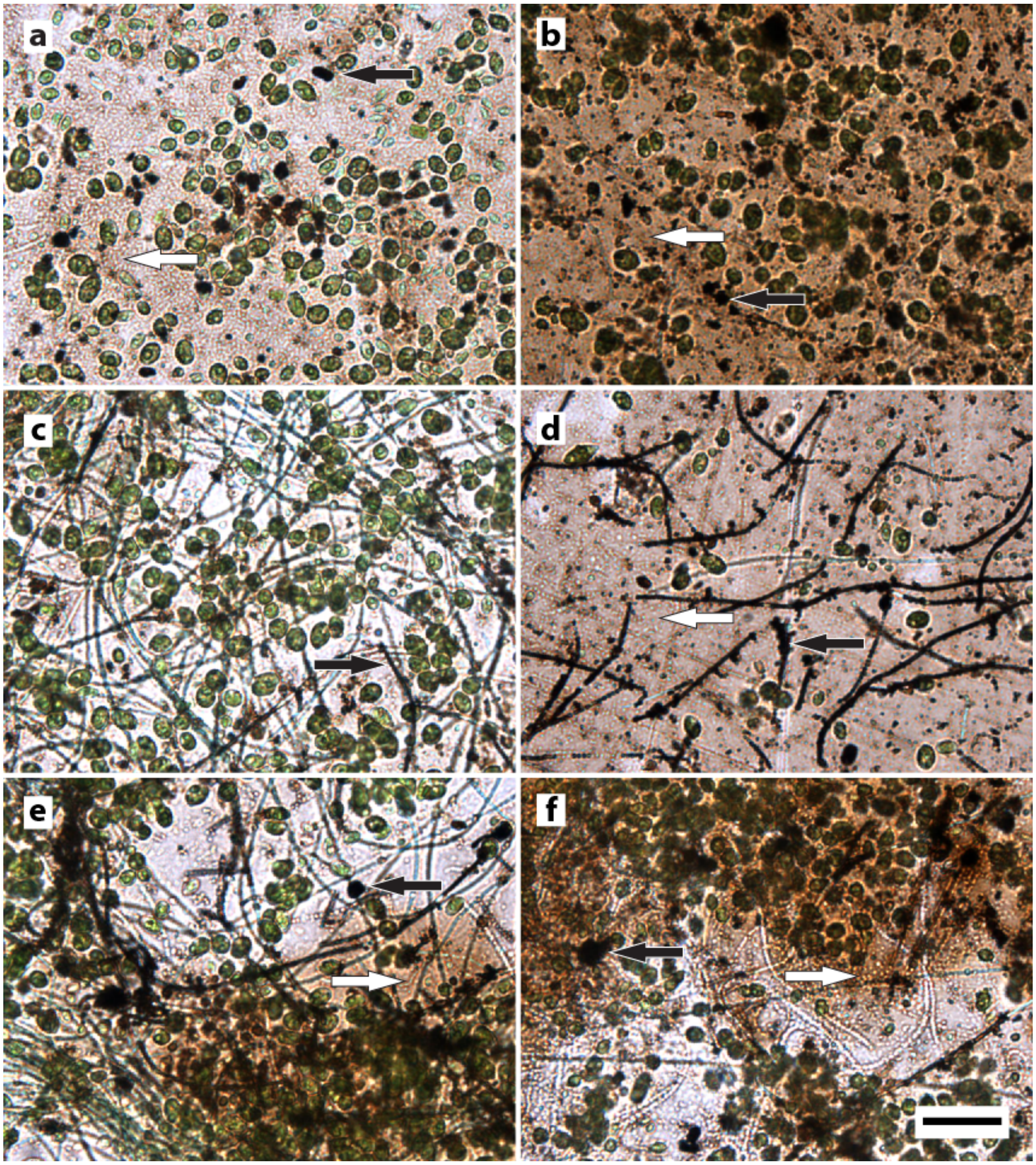

Figure S2. Mn(II) oxidation by mixed phototroph biofilm growing on a glass slide submerged in culture media containing 200 μM Mn(II). Phototrophs appear green or blue and Mn oxides appear brown/black under transmitted light. Scale bar is 30 μm. White arrows indicate diffuse oxidation throughout the biofilm, and black arrows indicate cell wall-associated oxidation.

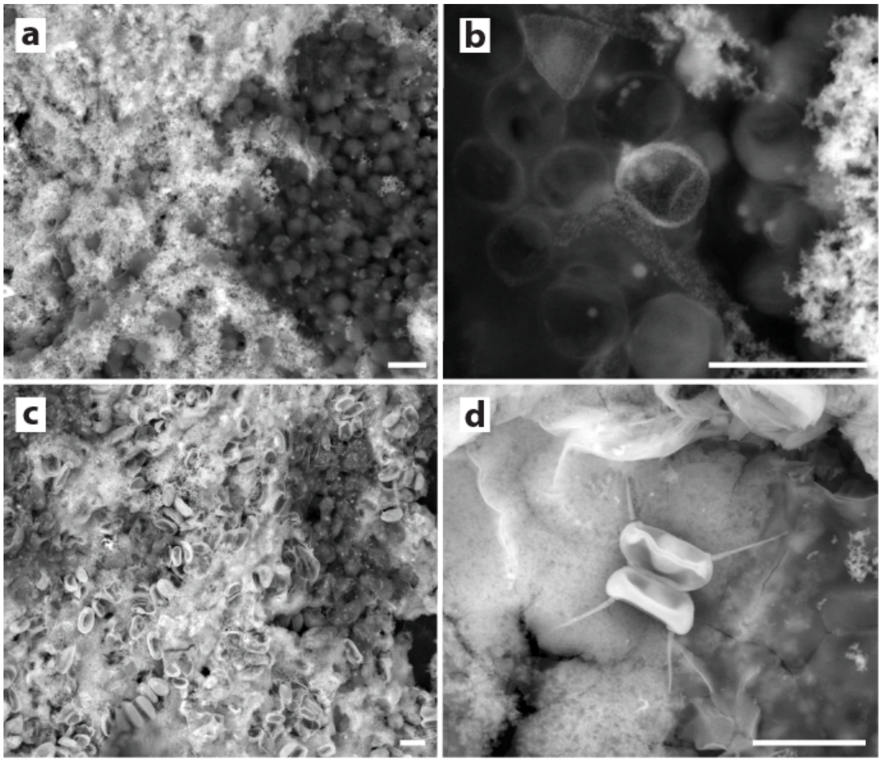

Figure S3. SEM of Mn(II)-oxidizing green algal isolates. Mn oxides appear white due to their higher molecular weight (compared with biomass). Scale bar is 5 µm in all images. (a) and (b) CM7-6.gr, identified as *Chlorella* sp., a single-celled, biofilm-forming green alga showing widespread biofilm-localised Mn(II) oxidation as well as occasional cell wall-associated oxidation after 37 days. (c) and (d) CM8-1.gr, identified as *Scenedesmus* sp., with extensive biofilm- and cell wall-associated oxidation after 37 days. Characteristic spines used for buoyancy and as possible protection against grazing are visible in (d).

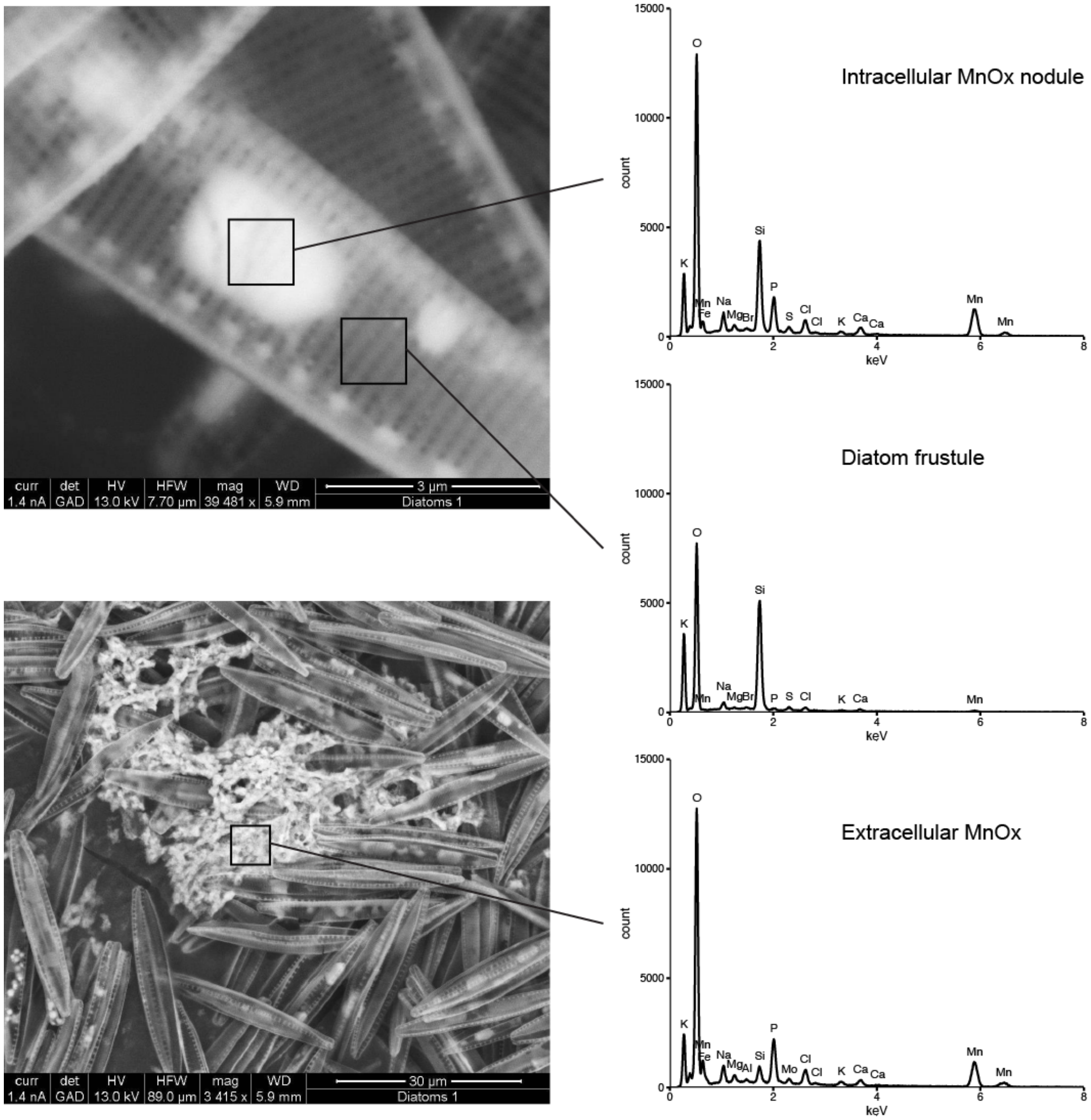

Figure S4. EDS spectra from different areas of diatom (isolate CM12-4.di) biofilm. Mn-rich areas are enriched in phosphorus.

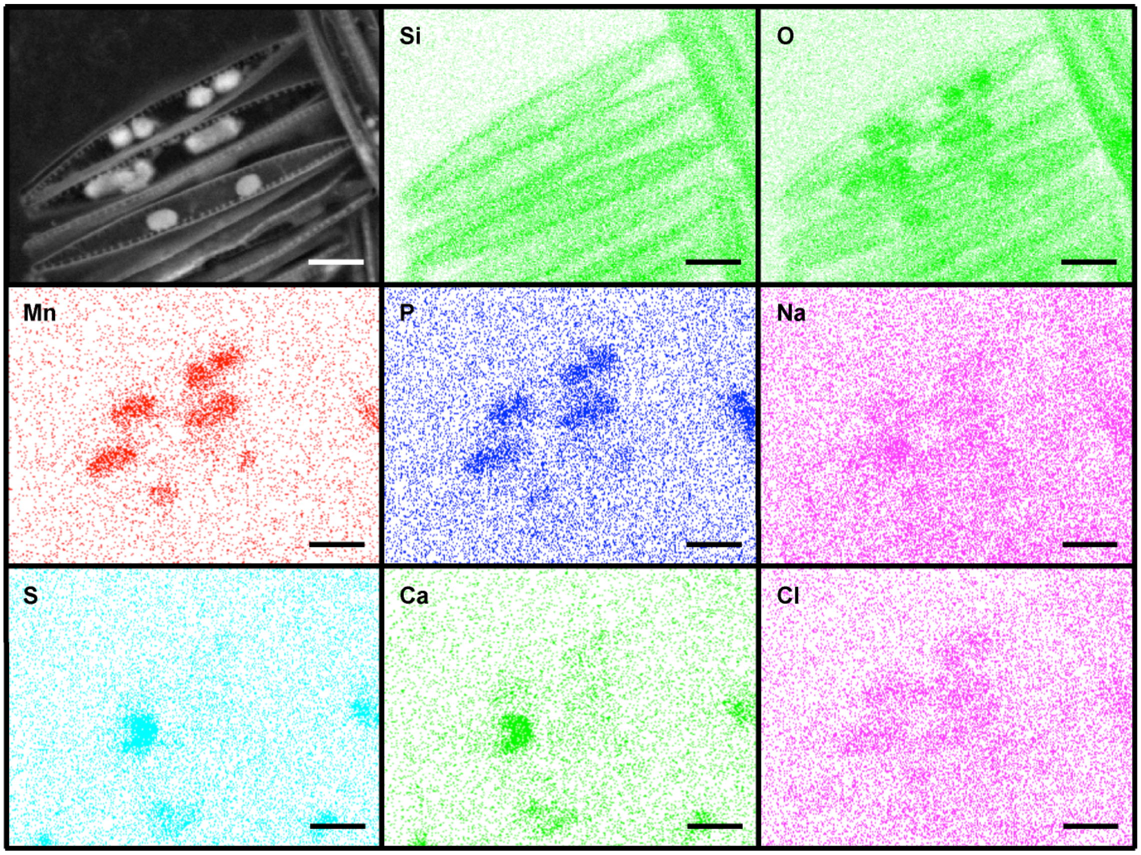

Figure S5. Element map of diatom CM12-4 with internal Mn-enriched nodules. SEM image is in the top left corner. Scale bar represents 5 µm. Phosphorus is enriched in the Mn nodules.

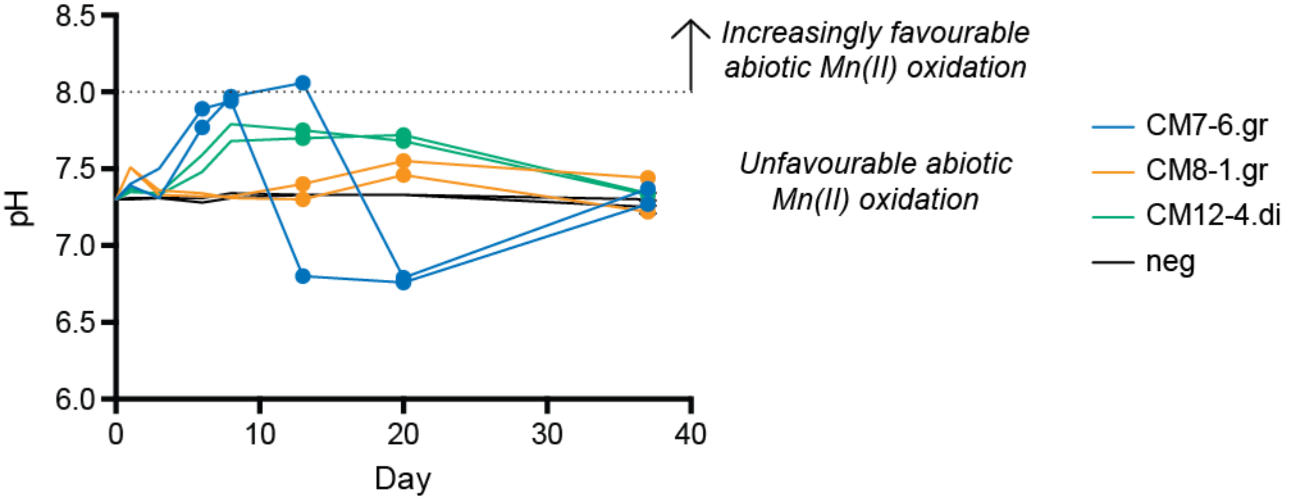

Figure S6. pH of duplicate cultures of algal isolates CM7-6.gr, CM8-1.gr and CM12-4.di over time. Culture media contained 200 µM Mn(II). Presence of Mn oxides is indicated by circles. Negative control consisted of sterile media only.

Table S1. Closest relatives of Mn(II)-oxidizing phototrophs isolated from CMD passive treatment systems, and relative abundance in culture-independent amplicon sequencing data [2]

Isolate	Gene fragment* (accession)	Sim. (%)	Closest relative in GenBank**		Relative abundance (%) in DS1 / DS2 sequencing data: ≥97% sim.	≥95% sim.
CM7-6	23S (MF278588)	100.0	KY629620	*Micractinium conductrix*	- / 0.19	0.10 / 0.58
	rbcL (MF278348)	97.0	MK295222	*Chlorella sp.* voucher FACHB-1531		
CM8-1	23S (MF278589)	99.1	GU939613	*Scenedesmus* sp. FSP-3	- / -	- / -
	rbcL (MF278351)	94.6	KT003376	*Desmodesmus spinosus* CCMA_UFSCar062		
CM8-2	23S (MF278590)	99.1	MH356727	*Seminavis robusta* D6	2.46 / 0.19	10.91 / 11.97
CM8-5	23S (MF278591)	100.0	DQ396875	*Scenedesmus obliquus* UTEX 393	- / -	- / -
	18S (MF278602)	100.0	AJ249515	*Scenedesmus obliquus* UTEX 1450		
	rbcL (MF278353)	99.5	KT777962	*Tetradesmus obliquus* SM17-1		
CM8-6	23S (MF278592)	96.3	EU725860	*Trebouxia asymmetrica* SAG 48.88	- / -	0.10 / 0.32
	rbcL (MF278344)	96.6	KY710860	*Oocystis nephrocytioides* CCALA 397		
CM9-5	rbcL (MF278350)	95.2	KT833563	*Kirchneriella obesa* CCMA-UFSCar 329	n/a***	n/a
CM9-6	rbcL (MG787874)	95.2	KT833565	*Kirchneriella lunaris* CCMA-UFSCar 423	n/a	n/a
CM11-1	23S (MF278593)	95.7	KM462883	*Geminella minor* SAG 22.88	- / -	- / -
	18S (MF278603)	93.6	KY006566	*Makinoella tosaensis* CCALA_961		
	rbcL (MF278345)	90.9	KF975594	*Ecballocystopsis* sp. AKS-4		
CM11-2	23S (MF278594)	100.0	KX507373	*Oedocladium carolinianum* UTEX LB 1686	1.45 / 0.13	1.45 / 0.13
	18S (MF278604)	99.2	DQ413048	*Oedogonium* sp. L3		
	rbcL (MF278342)	98.2	EU677193	*Oedogonium cardiacum* SAG 575-1b		
CM12-1	18S (MF278605)	94.6	FJ715936	*Hylodesmus singaporensis* E4-g	n/a	n/a
	rbcL (MF278352)	92.5	HQ246356	*Acutodesmus bajacalifornicus* ZA1-5		
CM12-4	23S (MF278595)	100.0	AP018511	*Nitzschia palea* NIES-2729	1.16 / 1.87	2.80 / 3.54
	18S (MF278606)	99.7	KY863476	*Nitzschia palea* TCC854		
CM12-5	23S (MF278596)	98.8	KJ718922	*Chlorella variabilis* NC64A	0.10 / 0.45	0.14 / 0.51
	rbcL (MF278347)	95.5	KJ718922	*Chlorella variabilis* NC64A		
MB7-1	23S (MF278597)	95.7	KM462883	*Geminella minor* SAG 22.88	- / -	- / -
	rbcL (MF278346)	90.5	KF975594	*Ecballocystopsis* sp. AKS-4		
MB7-4	23S (MF278598)	100.0	AP018511	*Nitzschia palea* NIES-2729	1.50 / 2.19	2.80 / 3.54
MBx9-1	23S (MF278958)	98.9	AM709632	*Pseudanabaena* sp. PCC 6903	- / 0.06	0.29 / 0.19
WC6-3	23S (MF278599)	95.7	L43353	*Chlamydomonas geitleri*	- / -	- / -
	18S (MF278607)	99.6	LC322161	*Chlamydomonas oviformis* SAG:11-20		
	rbcL (MF278343)	93.5	U80809	*Chloromonas* sp. ANT3		
WC7-3	23S (MF278600)	99.1	L43351	*Chlamydomonas agloeformis*	0.05 / -	0.97 / 0.64
	18S (MF278608)	100.0	AB490288	*Chlorococcum* sp. YF382		
WC8-1	23S (MF278601)	99.4	KM676717	*Oedogonium* sp. 1 AS-2014	1.45 / 0.13	1.45 / 0.13
	18S (MF278609)	98.6	KF569718	*Oedogonium* sp. PTP3		
	rbcL (MF278349)	97.2	DQ481215	*Oedogonium* sp. M8		

*23S refers to plastid 23S rRNA gene fragment, 18S refers to 18S rRNA gene fragment, rbcL refers to a fragment of the rbcL gene encoding the large subunit of RuBisCO (Ribulose-1,5-bisphosphate carboxylase/oxygenase)
**as of August 2019
***no 23S rRNA sequence available

Table S2. Culture media used in this study.

**Growth medium**	**AF6**	**BBM**	**Bristol**	**COMBO**	**MBL**	**WC**
Reference[a]	*NCMA*	*CSIRO*	*UTEX*	*NCMA*	*CSIRO*	*UTEX*
Reagent						
MES	2.05 mM					
$NaNO_3$	1.65 mM	2.94 mM	2.94 mM	1 mM	1 mM	1 mM
NH_4NO_3	27.5 μM					
$MgSO_4$	12.2 μM	304 μM	300 μM	166 μM	150 μM	150 μM
K_2HPO_4	73.5 μM	431 μM	430 μM	50 μM	50 μM	50 μM
KH_2PO_4	28.7 μM	1.29 mM	1.29 mM			
$CaCl_2$	68.0 μM	170 μM	170 μM	250 μM	250 μM	250 μM
Fe-citrate	8.2 μM					
citric acid	10.4 μM					
NaCl		428 μM	430 μM			
EDTA		428 μM				
KOH		1.38 mM				
$FeSO_4$		44.8 μM				
H_2SO_4		0.10%				
H_3BO_3		462 μM		16.2 μM		390 μM
$NaHCO_3$				150 μM	150 μM	150 μM
$Na2SiO_3$				100 μM	100 μM	100 μM
KCl				100 μM		
TRIS					2.1 mM	
Trace elements						
$FeCl_3$	3.63 uM			3.7 uM	11.7 uM	11.7 uM
$MnCl_2$	910 nM			910 nM	910 nM	910 nM
$ZnSO_4$	383 nM	76.7 uM		76.5 nM	76.5 nM	76.5 nM
$CoCl_2$	84.1 nM			42 nM	42 nM	42 nM
Na_2MoO_4	51.7 nM			24.8 nM	24.8 nM	26 nM
MoO_3		12.3 uM				
$CuSO_4$		15.7 uM		4.01 nM	4.01 nM	10 nM
$Co(NO_3)_2$		4.21 uM				
H_2SeO_3				12.4 nM		
Na_3VO_4				9.79 nM		98 nM
$ZnCl_2$						
Vitamins						
Thiamine	29.6 nM			296 nM	296 nM	296 nM
Biotin	8.2 nM			2.05 nM	2.05 nM	2.05 nM
Cyanocobalamin	0.738 nM			0.406 nM	0.369 nM	0.369 nM

Pyridoxine	5.91 nM					
pH	6.6	6.6		7.8	7.2	7.8
$MnCl_2$	200 µM	200 µM	200 µM	200 µM	200 µM	200 µM

[a]NCMA = National Center for Marine Algae and Microbiota (http://ncma.bigelow.org), UTEX = The Culture Collection of Algae at the University of Texas at Austin (https://utex.org), CSIRO = http://www.marine.csiro.au/microalgae/methods

Table S3. PCR primers used in this study

Target	Primers	Reference
Phototroph 23S rRNA	AlgaeF: GGACAGAAAGACCCTATGAA<br>AlgaeR: CCTGTTATCCCTAGAG	Sherwood and Presting[5]
Eukaryote 18S rRNA	EK-82F: GAAACTGCGAATGGCTC<br>EK-1520R: CYGCAGGTTCACCTAC	López-García et al.[6]
rbcL	rbcL 7F: CCAMAAACWGAAACWAAAGC<br>rbcL803: TCGTGCATAATAATAGGTACAC<br><br>rbcL320: TATTCGAAGAAGGTTCAGTAAC<br>rbcL1391R: TCTTTCCAAACTTCACAAGC	Nozaki et al.[7], Verbruggen et al.[8]

SUPPLEMENTARY REFERENCES

1. Luan, F., Santelli, C. M., Hansel, C. M. & Burgos, W. D. Defining manganese(II) removal processes in passive coal mine drainage treatment systems through laboratory incubation experiments. *Appl. Geochemistry* **27**, 1567–1578 (2012).
2. Chaput, D. L., Hansel, C. M., Burgos, W. D. & Santelli, C. M. Profiling microbial communities in manganese remediation systems treating coal mine drainage. *Appl. Environ. Microbiol.* **81**, 2189–2198 (2015).
3. Santelli, C. M. *et al.* Promotion of Mn(II) oxidation and remediation of coal mine drainage in passive treatment systems by diverse fungal and bacterial communities. *Appl. Environ. Microbiol.* **76**, 4871–4875 (2010).
4. Krumbein, W. E. & Altmann, H. J. A new method for the detection and enumeration of manganese oxidizing and reducing microorganisms. *Helgoländer Wissenschaftliche Meeresuntersuchungen* **25**, 347–356 (1973).
5. Sherwood, A. R. & Presting, G. G. Universal primers amplify a 23S rDNA plastid marker in eukaryotic algae and cyanobacteria. *J. Phycol.* **43**, 605–608 (2007).
6. López-García, P., Rodríguez-Valera, F., Pedrós-Alió, C. & Moreira, D. Unexpected diversity of small eukaryotes in deep-sea Antarctic plankton. *Nature* **409**, 603–607 (2001).
7. Nozaki, H. *et al.* Phylogenetic relationships within the colonial Volvocales (Chlorophyta) inferred from rbcL gene sequence data. *J. Phycol.* **31**, 970–979 (1995).
8. Verbruggen, H. *et al.* A multi-locus time-calibrated phylogeny of the siphonous green algae. *Mol. Phylogenet. Evol.* **50**, 642–653 (2009).
9. Benson, D. A., Karsch-Mizrachi, I., Lipman, D. J., Ostell, J. & Sayers, E. W. GenBank. *Nucleic Acids Res.* **37**, D26-31 (2009).
10. Altschul, S. F., Gish, W., Miller, W., Myers, E. W. & Lipman, D. J. Basic local alignment search tool. *J. Mol. Biol.* **215**, 403–10 (1990).
11. Estes, E. R., Andeer, P. F., Nordlund, D., Wankel, S. D. & Hansel, C. M. Biogenic manganese oxides as reservoirs of organic carbon and proteins in terrestrial and marine environments. *Geobiology* **15**, 158–172 (2017).
